# Mechanisms of fast and stringent search in homologous pairing of double-stranded DNA

**DOI:** 10.1371/journal.pcbi.1005421

**Published:** 2017-03-03

**Authors:** Amir Bitran, Wei-Yin Chiang, Erel Levine, Mara Prentiss

**Affiliations:** 1 Department of Physics, Harvard University, Cambridge, Massachusetts, United States of America; 2 FAS Center for Systems Biology, Harvard University, Cambridge, Massachusetts, United States of America; Weizmann Institute of Science, ISRAEL

## Abstract

Self-organization in the cell relies on the rapid and specific binding of molecules to their cognate targets. Correct bindings must be stable enough to promote the desired function even in the crowded and fluctuating cellular environment. In systems with many nearly matched targets, rapid and stringent formation of stable products is challenging. Mechanisms that overcome this challenge have been previously proposed, including separating the process into multiple stages; however, how particular *in vivo* systems overcome the challenge remains unclear. Here we consider a kinetic system, inspired by homology dependent pairing between double stranded DNA in bacteria. By considering a simplified tractable model, we identify different homology testing stages that naturally occur in the system. In particular, we first model dsDNA molecules as short rigid rods containing periodically spaced binding sites. The interaction begins when the centers of two rods collide at a random angle. For most collision angles, the interaction energy is weak because only a few binding sites near the collision point contribute significantly to the binding energy. We show that most incorrect pairings are rapidly rejected at this stage. In rare cases, the two rods enter a second stage by rotating into parallel alignment. While rotation increases the stability of matched and nearly matched pairings, subsequent rotational fluctuations reduce kinetic trapping. Finally, in vivo chromosome are much longer than the persistence length of dsDNA, so we extended the model to include multiple parallel collisions between long dsDNA molecules, and find that those additional interactions can greatly accelerate the searching.

## Author summary

Protein folding and the binding of sequence dependent proteins to DNA are examples of self-assembling systems in which the binding energy varies continuously throughout the interaction. Previous theoretical work has highlighted the importance of dividing the interaction into separate stages characterized by interaction times and binding energies that vary by orders of magnitude. Insight into how such a division might naturally arise and promote accurate and efficient self-assembly is provided by our study of a simple tractable model inspired by the homology dependent pairing of double stranded DNA molecules in vivo. In the model, the binding energy is controlled by one single continuously tunable variable whose natural evolution creates stages that efficiently and accurately form stable products.

## Introduction

Homologous pairing of DNA molecules is involved in many fundamental biological processes, including homologous recombination in meiosis, interaction between alleles on homologous chromosomes (transvection) [1], and homologous repair of double strand breaks [2]. Recent experiments have shown that dsDNA fragments in solution are capable of pairing in a homology-dependent manner even in the absence of proteins [2–8]. This protein-free mode of pairing, which can also occur in the presence of nucleosomes [7], is robust to salt concentration, PH, and shear force, suggesting that it may serve as the ‘default’ mode of chromosome pairing *in vivo* [2]. Various models have been proposed to explain the homology-dependent attraction between dsDNA molecules [9–11], many of which attribute this interaction to hydrophobic forces or electrostatics. Molecular dynamics simulations [11] show that adsorbed positive ions lie in the grooves of DNA molecules, which may suggest an attractive dipole-dipole attraction. While possible origins of attractive interactions have been considered, the kinetics of this pairing process have not been studied.

The homologous pairing of dsDNA molecules is one example of a biological system in which two molecules spontaneously attach to each other as a result of attractive interactions between multiple matching binding sites. Other *in vivo* examples include sequence-dependent DNA or RNA binding by proteins, target binding by small regulatory RNAs, and gene editing by CRISPR/Cas9. Such pairing processes face at least three key demands. First, they must form a product within a biologically reasonable timescale (speed). Second, they must form a product that is durable enough to perform a subsequent function (stability). Third, the error rate must be acceptably low for the given system (stringency) [12]. However, in systems where recognition involves many binding sites that contribute collectively to the interaction, the tradeoff between parameters can be incompatible with system requirements. This incompatibility has been referred to as the speed-stability-stringency (SSS) paradox [12–17].

Previously it has been shown that the tradeoff between speed and stability can be mitigated if the testing process is divided into multiple steps, characterized by different binding energies [14, 15]. Additionally, mechanisms of kinetic proofreading can allow such systems to achieve arbitrarily good stringencies [18, 19], at the expense of searching speed. In general, the tradeoff between speed, stability, and stringency depends strongly on the environment in which the search is performed, which includes the prevalence of near matches in the sample being searched, and the average time between collisions. The effectiveness of search strategies is also influenced by specific details of the interaction, such as the binding energies of matching sites and the characteristic decay length of the binding interaction.

Here, we investigate how a feature that is intrinsic to any collision between DNA duplexes–their freedom to rotate following a collision–affects the dynamics of homologous pairing. The rotational degree of freedom provides a continuum of testing stages characterized by different effective binding energies and interaction times. By analyzing an effective tractable model, we show that the rotational degree of freedom mitigates tradeoffs between speed, stability, and stringency as compared to a system in which the duplexes are rotationally constrained so that they remain aligned. This new result provides insight into dynamics of dsDNA-dsDNA pairing *in vivo*, and its general features may extend to other biological systems that depend on the pairing of matched binding sites.

## Models

We make a few key assumptions inspired by dsDNA-dsDNA pairing *in vivo*. First, we note that the crowded environment of the cell ensures that different regions of the genome collide frequently. We model each collision as an opportunity for homology testing. We assume that binding of homologous dsDNA regions promotes some function that is performed by a molecular machinery, provided that pairing of the two dsDNA regions is sufficiently persistent. We ask how long it takes for two homologous regions in a bacterial chromosome to find each other and stay bound long enough for that machinery to act. We model this action as an irreversible transition that makes the pairing permanent. RecA mediated homologous repair in bacteria may be an example of such a process. [20].

Theoretical work that describes homology dependent pairing of dsDNA highlight the importance of the helical nature of these molecules [9, 10, 21, 22]. The helical structure limits the interaction sites to roughly a few bases per helical turn, creating an interacting system in which discrete periodically spaced binding sites are separated by non-interacting zones. We therefore model a collision between two regions of bacterial chromosomes as a local interaction between two rigid ‘rods’ carrying a linear array of equally spaced binding sites. The binding sites on the rods are separated by 3.4 nm, on the assumption that two dsDNA molecules interact roughly once per helical turn. The total length of the rods is 17 binding sites or 57.8 nm, which is comparable to measured values for the persistence length of dsDNA. The energy associated with interaction between the two rods is given by the sum of interactions among their binding sites, which is taken to be exponentially decaying with distance. Thus, in this model the interaction energy between the two molecules depends only on two degrees of freedom: the planar angle between the two rods, and the distance between their centers. Together, these assumptions simplify the analysis of this model considerably, providing clear results that are easy to interpret.

**Fig 1:**
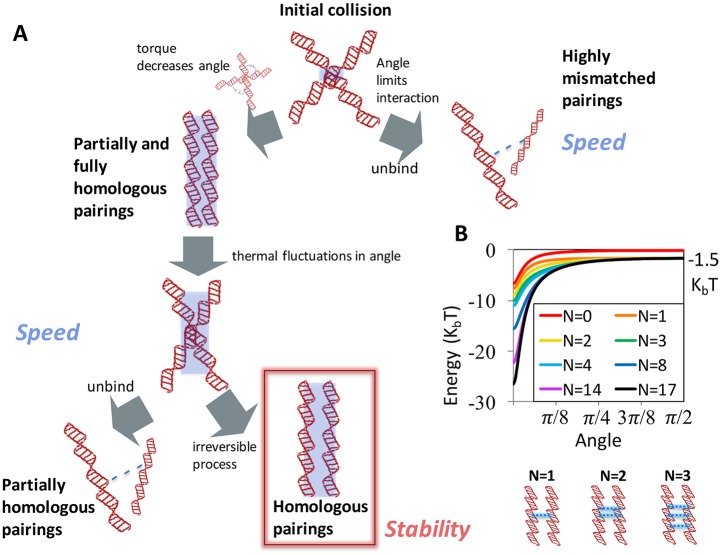
Model for interactions between dsDNA molecules. (A) Schematic of our model for multi-stage kinetic proofreading. Two DNA molecules (modeled as rigid rods) initially collide at a finite angle. The angle limits contact between interaction sites and ensures that highly mismatched pairings rapidly unbind irreversibly. Meanwhile, pairings with roughly 4 or more matching sites flanking the collision site experience an attractive torque that draws them into a more deeply bound, parallel state. Partially homologous pairings that reach this deeply bound state are destabilized by diffusive fluctuations in angle, which allow pairings to re explore larger angles, from which point irreversible unbinding is faster. These fluctuations provide further speed by allowing a given stringency to be attained faster. Finally, pairings that remain bound for a minimum amount of time are irreversibly stabilized, ensuring stability for homologs. In this diagram, the blue shading indicates the approximate length over which pairings interact. (B) Energy as a function of angle for pairs of rods with a variable number of continuous matched sites flanking the collision point, *N*. Example diagrams of pairings with *N* = 1, 2, and 3 are shown below the plot, with the blue, dashed lines indicating attractive interactions between matched pairs of sites. The attractive energy per matched site is -1.5 *k*_*B*_ *T*, marked on the plot.

Our simple model is illustrated in Fig 1. We define *θ* as the angle between the rods. We assume that a homology test begins with a collision between two molecules at an angle *θ*_0_. Because the spacing between binding sites is much larger than the short range of the interactions between binding sites, even a small *θ* limits the interaction to only a few bases near the interaction point (Fig 1B). We analyze this model within the framework of a discrete state Markov model. Thermal fluctuations allow *θ* to grow and shrink, but can also result in a complete and irreversible unbinding. Conversely, if the two molecules remain bound long enough, an external energy-driven process stabilizes the binding or executes downstream processes. For simplicity, we assume that this happens a fixed time *T*_*D*_ post collision, although other choices which provide a time delay can be considered. Our model does not allow for sliding of the two molecules with respect to each other. Assuming that sliding occurs on time scales that are significantly slower than rotation, such an event is captured by our model as a composition of unbinding and rebinding events. A detailed description of the model is provided in the SI.

In what follows, we consider the search process from a standpoint of an individual genomic locus, or a single rod searching for a homologous partner. At the end of the paper we extend our model to include the multiple parallel interactions that are characteristic of collisions between chromosomes, which are much longer than the dsDNA persistence length.

## Results

### Initial collision angle rapidly filters out mismatches

Limiting initial interactions to a few binding sites helps systems mitigate the SSS paradox by allowing mismatched pairings to rapidly unbind from a weakly bound state; however, that mitigation is most effective if the weak initial interaction can be followed by a homology dependent transition to a more deeply bound state that stabilizes the binding of correct pairings [12–14]. In what follows, we show that the rotational degree of freedom provides both of these features. In particular, a finite collision angle *θ*_0_ limits the number of binding sites that can interact strongly, resulting in a weak initial binding; however, attractive interactions between matched pairings exert a torque that induces rotation toward a parallel alignment. This rotation brings binding sites closer together, thus increasing the binding energy and enhancing the stability of correct pairings.

The challenge of finding a correct pairing depends strongly on the level of similarity among molecules in an ensemble. For concreteness, we define the ensemble by assuming that sequences are generated randomly from an alphabet of 1/*q* letters. Thus, two sites in two different sequences ‘accidentally’ match each other with probability *q*. With respect to a specific searching sequence, we group all other sequences into disjoint classes defined by the number *N* of consecutive matching sites around the midpoint, assumed to be the collision point between the molecules.

**Fig 2:**
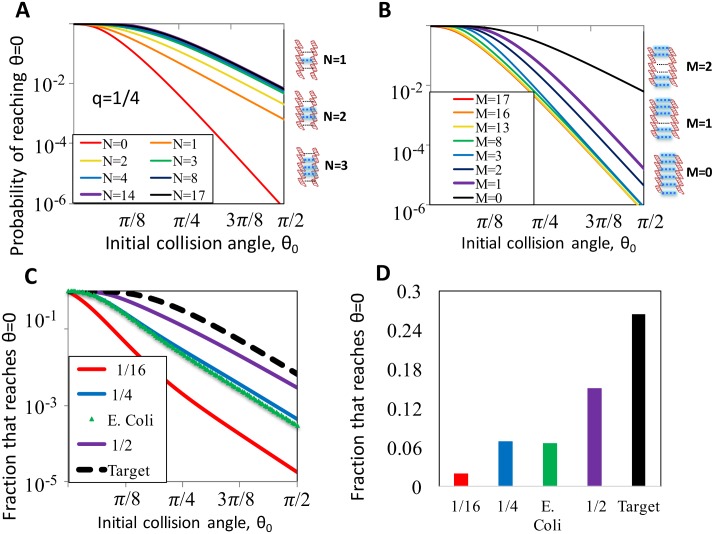
Transition from collision at random angle to parallel alignment. (A) Probability of reaching a parallel alignment as a function of the initial collision angle *θ*_0_ and the number of continuous matched sites flanking the collision point *N*. Here *q* = 1/4. (B) Same as (A), for sequences where *M* sites flanking the collision point are the only ones *not* forced to match. (C) Fraction of attempted pairings that reach a parallel alignment as function of initial collision angle. Solid lines, mismatched random sequences with *q* = 1/16, 1/4, or 1/2, and with the *E. coli* genome statistics. Dashed line, perfectly matching sequences. (D) The results of (C) averaged over collision angles up to *π*/2, assuming collisions are uniformly distributed in 3d.

The kinetics of the interaction between two rods depend on both *N* and *q*. In Fig 2A we show *p*_*N*_, the probability that a collision at initial angle *θ*_0_ leads at some point to perfectly parallel molecules (*θ* = 0) for different values of *N* and for *q* = 1/4. For each *N* we considered 2000 different randomly chosen sequences and averaged over the results for all of them. With a smaller *q* the number of ‘accidentally’ matching sites beyond the given *N* is expected to be smaller, and therefore rejecting these sequences is expected to be easier (Fig 2A and S1 Fig).

Our results show that without requiring any matches near the collision site (*N* = 0), the probability of rotating into a parallel alignment decays very rapidly with *θ*_0_. This decay becomes slower already with a single match between the molecules (*N* = 1), a behavior that persists for *N* = 2, 3 and saturates at *N* = 4. This suggests that mismatched pairings are likely to be rejected quickly, while matched sites immediately around the collision points contribute significantly to stabilization.

Importantly, the probability of proceeding from the initial binding to a parallel configuration is acutely sensitive to mismatches around the collision point, even when those mismatches are embedded in a rod that otherwise perfectly matches its target, as shown in Fig 2B. For *θ*_0_ > *π*/8, even a sequence with one mismatched binding site (*M* = 1) has significantly lower probability of reaching parallel alignment than a perfectly matched sequence (*M* = 0). Increasing the number of mismatched bases further reduces the probability that parallel alignment will be obtained.

To place the effect of the initial collision angle in the context of the entire search process, we calculate the probability that any collision would lead to a parallel configuration as the weighted average *P* = Σ_*N*_ *f*_*N*_ *p*_*N*_ of the interactions characteristic of dsDNA collisions in the sample. Here *p*_*N*_ is again the probability that a pair with *N* matches near the collision point reaches a parallel alignment (Fig 2A), and *f*_*N*_ is the normalized frequency of pairs with *N* such matches in the ensemble of targets (S2 Fig). In the context of a target search on the chromosome, this ensemble contains all possible segments of the prescribed length found in the genome of that organism. For a random set of targets, this ensemble is characterized by a single parameter *q* defined above, which is simply the inverse of the number of possible types of binding sites. In Fig 2C we plot the probability *P* for several values of *q*, as well as for the ensemble defined by the *E. coli* genome. Comparing with the same probability for a perfect pairing (dashed line), it is clear that the advantage of the initial step is more significant when accidental matches are more rare. This effect persists for all collision angles.

In Fig 2D, we show the probabilities of reaching *θ* = 0 assuming that collision angles are uniformly distributed over a half-unit sphere, *p*(*θ*_0_) ∼ sin(*θ*_0_). We obtain these values by averaging the traces in Fig 2C from *θ* = 0 to *θ* = *π*/2. This figure summarizes the main result of this section, that the transition from initial collision to parallel configuration rapidly rejects many mismatches. In the next section, we consider how speed and stringency are influenced by proofreading steps after a parallel alignment is reached.

### Rotational fluctuations attenuate kinetic trapping

Although the period from initial binding to arrival at a parallel alignment screens against many mismatched pairings, some of these pairs will reach this deeply-bound configuration. Kinetic trapping, whereby almost-matched sequences remain bound for a significant time before they unbind and resume the search, can lead to an appreciable slowdown in the search process, especially in an ensemble that contains many similar sequences. However, as we show next, rotational fluctuations can provide a transient decrease in the unbinding barrier that reduces kinetic trapping. The reduction in kinetic trapping occurs because rotational fluctuations transiently decrease the binding energy, by increasing the separation between corresponding binding sites.

**Fig 3:**
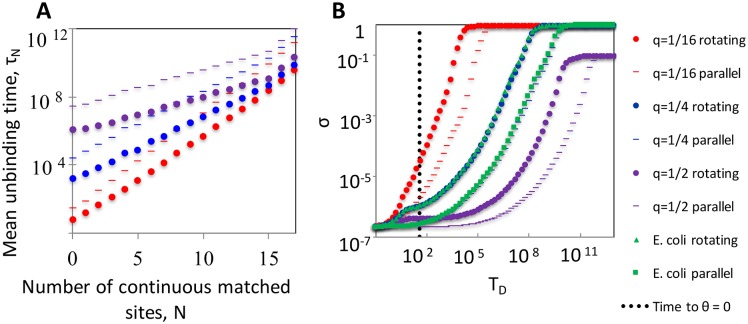
Rotational fluctuations mitigate kinetic trapping. (A) Mean unbinding time for pairs of rods with *N* continuous matched sites about the center. We consider both rotating rods that begin at *θ*_0_ = 0, and constrained parallel rods that are allowed to unbind but not rotate. Three accidental match probabilities are considered for sites beyond *N*. All times in this figure are measured in units in which the rate of unbinding equals 1. (B) The specificity *σ* as a function of the delay time between collision and irreversible binding. The mean time required for a homologous pairing (with *q* = 1/4) to rotate to parallel alignment is marked reference (dashed line).

To demonstrate this, we set *θ*_0_ = 0 and calculate the mean unbinding time as a function of *N* when rotational fluctuations are present, and compare the results with the case where *θ* = 0 at all times. Fig 3A shows the mean unbinding times as a function of *N* for various values of *q*. In all cases, rotational fluctuations accelerate unbinding considerably. In particular, even when the two molecules are very similar (*N* near 17) the unbinding occurs around 100 times faster when the molecules are allowed to rotate around the collision point (S3 Fig). For *q* = 1/4 and *q* = 1/2, rotational diffusion offers a significant speed advantage even at low *N* values, as we discuss below. We note that the remaining *q* dependence of the results at high *N* comes mostly from interactions from sites in the two molecules that are not directly facing each other.

The impact of rotational fluctuations is related with the range of angles that are visited by the two rods before unbinding. If the energy barrier for unbinding is high enough (as it is for large *N*) or if the time scale associated with these fluctuations are fast, the system achieves a quasi-equilibrium state where the distribution of angles is given approximately by the Boltzmann distribution (S4 Fig). In this case, the two rods spend significant time in relatively wide angles, where unbinding is more likely. Conversely, if the barrier for unbinding is not high (such as for small *N*), unbinding is likely to occur before arrival at significant angles, and the difference between the rotating and frozen systems is diminished.

### Rotational fluctuations reduce the search time required to achieve high stringencies

Given these results, which suggest that rotational fluctuations can accelerate unbinding of unwanted pairings, we turn to the effect of these fluctuations on the stringency of the search process, quantified by the error rate in the irreversibly paired products. In what follows, we show that the rotational degree of freedom allows high stringencies to be achieved orders of magnitude faster than they can be reached in the rotationally constrained system where *θ* = 0 at all times.

Consistent with well-known properties of kinetic proofreading systems, increasing the time delay, *T*_*D*_, between the initial binding and the irreversible stabilizing process increases the stringency; however, the introduction of such a time delay slows down the search process because increasing *T*_*D*_ also reduces the probability *P*_*T*_ that a pairing with the true target become irreversibly bound. If a correct pairing unbinds, the search process must start again.

We define the achieved *specificity* *σ*(*T*_*D*_) as the probability that a searching sequence ultimately binds to it homologous sequence. This probability is given by

$$\sigma \left(T_{D}\right)=\frac{f_{T}P_{T}\left(T_{D}\right)}{f_{T}P_{T}\left(T_{D}\right)+\sum_{N=1}^{17}f_{N}P_{N}\left(T_{D}\right)}\;,$$ (1)

where *f*_*T*_ is the frequency of target sequences (assumed to be 1 in the ensemble) and *f*_*N*_ is the frequency of target sequences containing *N* accidental matches (S2 Fig). Here again *P*_*T*_(*T*_*D*_) is the probability that pairing with the true target remains bound at time *T*_*D*_, and *P*_*N*_(*T*_*D*_) is the same probability for an off-target with *N* continuous matched sites. Alternatively, the outcome of the search could be quantified in terms of the error rate *η*(*T*_*D*_), defined as the probability that a searcher is bound to an off-target sequence at time *T*_*D*_, which is related with the specificity by *η*(*T*_*D*_) = 1 − *σ*(*T*_*D*_).

Fig 3B shows the specificity as function of *T*_*D*_ in both the rotating system (averaged over the collision angle as in Fig 2D) and the rotationally-constrained model. Results are shown for three values of *q*, as well as for the empirical distribution of accidental matches in the *E. coli* genome. The characteristic time to reach a parallel configuration for *q* = 1/4 is plotted for reference. Smaller values of *q* allow higher specificity to be attained faster as mismatched pairings with less accidental matches tend to unbind more quickly. This figure demonstrates that a much shorter time delay is required to achieve a certain level of specificity if rotational fluctuations are allowed. For example, to obtain *σ* > 99%, the rotationally-constrained system requires a *T*_*D*_ that is approximately 100 times larger than rotating system. Note that the *q* = 1/2 case cannot achieve *σ* = 1 because on average a bacterial genome contains ∼ 40 sequences that match the 17 consecutive binding sites in the searching sequence. In our model, these sequences cannot be distinguished from the true homologous partner.

The fact that a smaller time delay *T*_*D*_ suffices to guarantee a required specificity has a strong impact on the overall search time of the target. In general, the search period can be broken into periods punctuated by encounters with the true targets. Since the probability that such an encounter ends in irreversible binding is *P*_*T*_, the mean number of such periods is 1/*P*_*T*_. In each period, the searcher spends time *τ*_off_ bound to off-targets, *τ*_diff_ in free diffusion, and *τ*_target_ interacting with the true target. Together

$$\left\langle T_{\text{search}}\left(T_{D}\right)\right\rangle =\frac{1}{P_{T}\left(T_{D}\right)}\left[\tau_{\text{off}}+\tau_{\text{diff}}+\tau_{\text{target}}\right]\;.$$ (2)

Since the number of off-targets is very large we generally expect *τ*_off_ ≫ *τ*_target_, and therefore neglect the latter.

To compute *τ*_off_, we assume that the searcher interacts with a set of targets that obeys the statistics of the entire ensemble (this might not be the case *in vivo*, as crowding in the cell may limit the searcher to a local environment in the genome, that could have its own statistical properties). Under this assumption, we have

$$\tau_{\text{off}}=\int_{0}^{\theta_{\max}}d\theta_{0}\;p\left(\theta_{0}\right)\sum_{i=1}^{17}f_{N}\tau_{N}\left(\theta_{0}\right)\;,$$ (3)

where *τ*_*N*_(*θ*_0_) is the mean unbinding time for a pairing with *N* consecutive matches starting from a collision at angle *θ*_0_. Here we allow the possibility that the collision angle between two fragments of the genome is bounded by some *θ*_max_ < *π*/2 due to molecular crowding in the cell, as discussed below.

In what follows, we let *f*_*N*_ take the frequencies found in the *E. coli* genome. To compute *P*_*T*_(*t*), we choose *T*_*D*_ as the minimum time required to achieve a specificity equal to 0.99 of the maximum attainable value. We start our discussion by neglecting the time spent in free diffusion (i.e. set *τ*_diff_ = 0), and come back to it at the end of the section.

**Fig 4:**
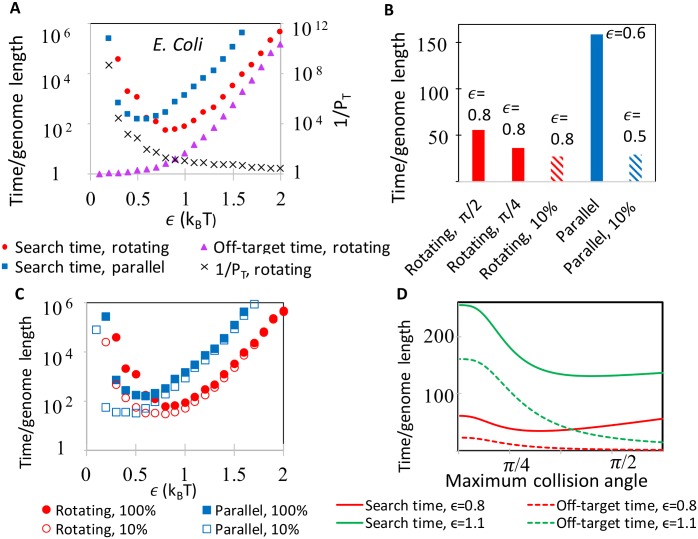
Determinants of total search time. (A) Kinetics of search as a function of *ϵ*, the attractive energy per matched site. Mean search time for a freely rotating searcher (red) and constrained parallel searcher are compared with the average interaction time with off-target sequence per search cycle for freely-rotating searcher (purple). Also shown is the mean number of pairing attempts, 1/*P*_*T*_. Here *τ*_diff_ = 0, *θ*_max_ = *π*/2, and genome statistics taken from the *E. coli* genome. All times in this figure are measured in units in which the rate of unbinding equals 1. (B) Minimum attainable homology search times. Indicated are the *θ*_max_ (*π*/2 when not indicated), the required specificity (100% if not indicated), and the value of *ϵ* at which these minima occur. (C) Comparison between the search time with *T*_*D*_ set to guarantee perfect specificity(*σ* = 1, as in panel B), and when it is set such that *σ* = 0.1 (empty symbols). (D) Homology search time (solid lines) and average off-target interaction time (dashed lines) for a freely rotating searcher as a function of *θ*_max_.

The search time is highly dependent on the amplitude *ϵ* of the pairwise site interaction energy (Fig 4A, S5 and S6 Figs). Small *ϵ* lessens the binding energy difference between the true target and its close matches, thus reducing stringency; it also increases the likelihood of unbinding before forming an irreversible product. Small *ϵ* therefore increases the search time by increasing the delay time *T*_*D*_ required to achieve a given stringency, and by increasing the average number binding attempts 1/*P*_*T*_ required before an irreversible product is formed. The increase in 1/*P*_*T*_ results from two effects: decreasing *ϵ* lowers the probability that the rods will rotate to *θ* = 0, and increasing the likelihood of unbinding in cases where parallel alignment is achieved. Overall, when *ϵ* is small the rotational degree of freedom increases the search time, while when *ϵ* is large, and kinetic trapping impedes the search, rotations can substantially reduce the search time. Importantly, the minimum search time as a function of *ϵ* is smaller in the rotating system than in the rotationally constrained system if high stringency is required (Fig 4B). In contrast, if low stringency is acceptable, the rotational degree of freedom may not reduce searching times (Fig 4C).

In the cell, crowding may weight the collision angle probability toward small angles. Such change in the distribution of the collision angle increases the probability of rotation into parallel alignment, which increase the probability that homologous pairings form irreversible products, but also reduces the fraction of mismatched pairings that are rejected immediately after collision. The balance between the two effects is demonstrated in Fig 4D, where we plot the mean search time for a different values of *θ*_max_, demonstrating that at larger *ϵ* the positive effect on kinetic trapping supersedes the negative effect of failing to catch the true target.

Finally, we note that the results of Fig 4 may underestimate the importance of the number of rounds of binding attempts by neglecting the diffusion time *τ*_diff_. In S7 Fig we show the search time as a function of *ϵ* for *τ*_diff_ = 1 and 100. As expected, increasing the diffusion time reduces the overall benefit due to rotation, because it magnifies the importance of increasing *P*_*T*_ over that of decreasing trapping. Thus, rotation is particularly beneficial if the system spends a limited amount of time diffusing, and the benefit is greatest if the diffusion time is much smaller than the time spent interacting with off-target sequences.

### The accelerating effect of parallel interactions

Above, we considered an interaction between the dsDNA to be limited to a region with a length of the order of the persistence length, so both dsDNA could be modeled as rigid rods that make contact at only one single point; However, bacterial genomes are much longer than the persistence length of dsDNA, so two copies of the same genome could make simultaneous contact at multiple positions. Thus, the interaction between the two regions of the genome may consist of multiple simultaneous binding attempts. If these *N* searches were uncorrelated, we would expect the search time to be reduced by a factor of 1/*N*. Further reductions in search time can be achieved if kinetic trapping can persist for times comparable with the entire search time, since a parallel search can continue even if one searcher is kinetically trapped [16].

However, the parallel searches that occur at multiple collision points between two segments of the genome are not uncorrelated, since the regions are physically connected. For some choices of search parameters, establishment of one correct pairing rapidly leads to the establishment of several correct pairings. As long as one of those correct pairings lead to an irreversible product before all of the pairings unbinding, the search time penalty due to the unbinding of correct pairing is greatly reduced without significantly reducing the efficiency with which incorrect pairings are rejected. This advantage mainly affects the freely rotating system, where rejections of true targets are more likely.

To get an insight for the contributions of multiple local collisions, consider the simple case where *k* searchers collide with their true target. The probability that *at least* one of them becomes irreversible bound, $P_{T}^{\prime }$, is given in terms of the probability *P*_*T*_ for successful collision such that

$$P_{T}^{\prime }\left(k\right)=1-{\left(1-P_{T}\right)}^{k}\;.$$ (4)

For example, choosing the delay time *T*_*D*_ such that *σ* > 0.99, we have *P*_*T*_ ≃ 0.2 but $P_{T}^{\prime }>90\%$ if the number of collision points is *k* > 20. This suggests a 4.5-fold reduction in the number of expected rounds of pairing attempts. Since this number is a main contributor to the search time at weak pairwise interactions (small *ϵ*), we find a significant decrease in the search time in this range (S8 Fig). Thus, parallel local searches extends the advantage of rotational fluctuations towards lower values of *ϵ*.

## Discussion

In our model the rotation of two molecules about their collision point represents a particular example of a degree of freedom that allows the effective interaction strengths to vary as binding progresses. Other examples may include the degree to which DNA is wrapped around histones, the relative orientation of paired histones and DNA, as well as the stem-loop RNA structures during small RNA-mRNA interactions. In this paper we assume that the pairwise interaction between binding site decays exponentially with distance; our conclusion however are not sensitive to the form of these interactions, as long as they decay rapidly (S9 Fig).

Although the binding energy varies continuously with *θ*, we have discussed the binding progression in terms of stages with different characteristic times and binding energies. As shown in Fig 3, the reversible interactions occur on timescales that typically vary by several orders of magnitude. In order of increasing duration, the major characteristic timescales are: (i) the characteristic interaction time for a collision involving completely mismatched partners (∼ 1); (ii) the characteristic time required for a matched interaction to rotate the colliding partners into parallel alignment (*τ*_rotation_ ∼ 100); and (iii) the characteristic unbinding time for near matches that have rotated into parallel alignment (>10^6^ × *τ*_rotation_), which dictates the choice of the waiting time *T*_*D*_ to irreversible transition.

A direct consequence of this time scale cascade can be tested experimentally. Consider a mixture of two types of short dsDNAs that contain 3 mismatched sites. If these sites are well-separated, all in registration collisions between the molecules will lead to quick rotation into parallel configuration. However, if the 3 mismatched sites are grouped together, collisions at these sites will be quickly rejected. The expected difference in the statistics of unbinding times, which can be measured e.g. using FRET, is a signature of the rotational degree of freedom.

Our system maps closely to many features of the protein/DNA recognition model proposed by Slutsky and Mirny [14], but our simplified model allows exact calculations of the binding energy over a continuum of binding states. We propose that the weak initial interaction in the protein system corresponds to the weak initial interaction that occurs when two rods collide at a significant angle. In addition, a conformational change of the protein leading to stronger DNA binding is analogous to homology-dependent rotation of the rods into parallel alignment. Furthermore, thermal fluctuations allow proteins that incorrectly undergo conformational change to change back and continue searching, just as thermally driven angular fluctuations destabilize rod pairings that incorrectly reach parallel alignment. Our model, however, distinguishes between the role of rapid initial screen, that occurs during the transition from an initial collision angle, and the extended interrogation of the target, that occurs once a parallel alignment is achieved. Finally, if high kinetic barriers block folding to deeply bound states, then the long times required to overcome the barriers may be analogous the long delay time *T*_*D*_ that precedes irreversible binding in the rotating system.

Our representation of dsDNA as a rigid rod with one binding site per helical turn may miss important features of dsDNA-dsDNA pairing *in vivo*, including protein binding, histone wrapping, the helical geometry of the genome, molecular crowding, and mechanical and entropic penalties due to pairing. Nevertheless, many of the key features of our model may still contribute to understanding how multiple separated protein-free regions of nucleosomal dsDNA pair rapidly and specifically, as we discuss in the following.

In general, interactions between matching genomic sections should be transient, since it is undesirable that genomic segments bind together permanently. This is consistent with our model of the first two stages in the pairing process. *In vivo*, the initial stages may almost always lead to dissolution of pairing rather than formation of an irreversible product; however, such transient interactions can be important if they preposition DNA for a subsequent interactions, including irreversible biochemical process such as RecA mediated repair of double strand breaks [20].

The relationship between our model and the function of RecA family proteins may extend beyond the protein’s providing a final irreversible step in the binding process. RecA protein family mediated homologous recombination may itself represent an example of an *in vivo* system that exploits features elucidated by the model presented in this paper. In the RecA system the final product depends on the binding between bases in one strand of a dsDNA molecule and bases in an ssDNA strand that is embedded at the center of a nucleoprotein filament formed when RecA binds to the ssDNA. In the context of recognition in the RecA system, we define a binding site as an ssDNA base. A product is formed when an ssDNA base in the filament pairs with an ssDNA base in one of the strands of the dsDNA. The ssDNA in the filament is extended by 1.5 x the B-form length, so homologous pairing requires that the dsDNA strand that pairs with the ssDNA also extend to 1.5× the B-form length. Seminal theoretical work has considered how the registration mismatch between B-form dsDNA and RecA bound ssDNA may influence homology recognition by limiting homologous contacts [23]. More recent theoretical work has considered how the extension of dsDNA that results when the dsDNA binds to RecA filaments may enhance homology recognition. That work showed that the free energy penalty due to the stretching of the bound dsDNA optimizes recognition in a system where the energy penalty was assumed to depend linearly on the number of base pairs bound to RecA [24]. Later theoretical work extended deGennes treatment of the shearing of dsDNA [25] to dsDNA bound to RecA and showed that the mechanical binding energy may include a term which is a non-linear function of the number bound dsDNA [26]. Recent molecular modeling supports the existence of such a non-linear term and indicates that the term may play a vital role in limiting the initial homology testing to 8 bp [27]. This initial 8 bp test can reject ∼ 95% of attempted pairings to unbind without further testing [28–30].

The dsDNA-dsDNA pairing considered in this work and the homology recognition mediated by RecA family proteins may share some important common features: (1) An initial interaction that limits contact between binding sites unless the initial interaction passes a homology test, in the dsDNA-dsDNA pairing case the limitation is due to the initial collision angle that creates a separation between binding sites and in the RecA case it is due to the structure of the bound dsDNA that creates a separation between binding sites. (2) Iterative homology dependent progression toward more deeply bound states. (3) A mechanism for catastrophic unbinding from fairly deeply bound states, which in the dsDNA-dsDNA system results from thermal fluctuations in the angle between the rods, but in the RecA system involves much more complicated and difficult to model interactions between the proteins and the DNA. (4) A low probability that even a correct pairing will progress to an irreversible state that is combined with simultaneous parallel testing of separated regions, which allows correct pairings to have a high probability of becoming irreversible even though each individual region has a low probability.

The dsDNA-dsDNA system and RecA protein family mediated homologous recombination also have some differences. In the RecA system, the DNA and the protein constantly restructure as recognition progresses and unfavorable mechanical energy terms that depend non-linearly on the number of extended dsDNA bases play important roles, making energy calculations very challenging. The simplified dsDNA-dsDNA model considered here does not allow any deformation of the rigid rods and there are no unfavorable mechanical energy terms. We note that recent theoretical work [31] includes the possibility that the real dsDNA-dsDNA system may include a non-linear free energy term due to the torque on the dsDNA that results because the helical period of dsDNA is not an integer multiple of the number of base pairs, so torsional deformation of the helix is required in order for successive helical turns to be aligned in registration. We have shown that this term is not required to achieve recognition, but future work may consider how the presence of a non-linear term alters the results that we observed under the assumption that the rods are not deformable.

In the case of the RecA system, during the initial interaction the geometry of the nucleoprotein filament limits the initial interaction between the incoming and complimentary strands to ≈ 8 bp whose binding is easily reversed, allowing ∼ 95% of attempted pairings to unbind without further testing [28–30]. The rare pairings that pass the initial test progress to more deeply bound states as more base pairs are allowed to interact. After more stable state bounds are reached, thermal fluctuations may promote catastrophic unbinding that reduces kinetic trapping [12]. Furthermore, in the RecA system correlated parallel testing may allow pairings that are homologous over >1000 bp to have a high probability of forming an irreversible product, even though the probability of irreversible pairing between ∼ 100 bp sequences is rather low [32].

Finally, parts of the model may provide insight into some of the function of other systems. For example, BRCA2 polymers with bound Rad51 may exploit multiple weak correlated parallel binding to differentiate between dsDNA and ssDNA targets [33], and synthetic polyvalent inhibitors may also exploit the same mechanism to improve specificity at low global concentrations [34, 35].

## Supporting information

S1 Fig Transition from collision at random angle to parallel alignment. Same as Fig 1(B) of main text, but with (A) *q* = 1/16, and (B) *q* = 1/2.
(TIF)

S2 Fig Statistics of target ensemble. *f*_*N*_, the expected abundance of sequences that will match a given sequence at *N* continuous sites surrounding the collision site. Random genomes with different values of *q* are compared with the *E. Coli* genome.
(TIF)

S3 Fig Homology dependence of rotation. Ratio of mean unbinding time for rotating rods to mean unbinding time for parallel rods as a function of *N*, the number of continuous matched sites, assuming the rotating rods begin at the initial state of *θ* = 0, for several accidental match frequencies *q*. The effect of rotational fluctuations is enhanced when the number of matches between the two sequences increases and the free-energy profile becomes steeper. This can happen either by imposing larger number of matches (larger *N*) or by more frequent accidental matches (larger *q*).
(TIF)

S4 Fig Angle fluctuations. (A) Shows the probability of rotating to an angle with a certain departure in energy relative to the energy value at *θ* = 0 (x axis) at various times after a collision. At each time, the probability is averaged over 2000 randomly generated sequences with N = 6 continuous matches about the center and *q* = 1/4. This distribution is plotted alongside the Botlzmann distribution, which is averaged over the same 2000 sequences. (B) Same as (A) with N = 17.
(TIF)

S5 Fig The effect of site-site interaction energy. Off-target time and total search time for freely rotating rods, and search time for constrained parallel rods as a function of *ϵ*, the attractive energy per matched site in units of *k*_*B*_ *T*. Collisions angles are assumed to be uniformly distributed in 3d between 0 and *π*/2 and *q* is set to 1/16. Values for 1/*P*_*T*_ are shown on the right vertical axis.
(TIF)

S6 Fig The effect of site-site interaction energy. Fraction of search time spent interacting with off-target sequences with *N* continuous matches as a function of *ϵ*, the attractive energy per matched site. At low *ϵ* the search time is dominated by pairings with low *N*. But as *ϵ* increases, kinetic trapping becomes more problematic. Thus, the off-target interaction time becomes dominated by species with higher *N* when *ϵ* is large. Genome statistics from the *E. Coli* genome are assumed.
(TIF)

S7 Fig Effects of diffusion time. (A) Search time as a function of *ϵ* for freely rotating and constrained parallel rods assuming a diffusion time *τ*_off_ of 1 (A) and 100 (B). Collision angles are averaged up to *π*/2 as in the main text, and genome statistics from the *E. Coli* genome are used. Increasing diffusion time penalizes the rotating system to a greater extent than the parallel system owing to the former’s low value of *P*_*T*_. Nonetheless, rotation is consistently beneficial above a minimum *ϵ* value, which increases along with *τ*_off_.
(TIF)

S8 FigParallelized search.(A-D) The effect of *k* genomic segments searching in parallel on the search time, as a function of *ϵ*. At low *ϵ*, *P*_*T*_ for a single searcher is very small due to the specificity requirement, and parallel searching is not useful. But at high energies, $R_{H}^{\prime }$ approaches 1 as *k* grows. Thus, both search times are decreased, particularly the rotating search time, which suffers from a worse *P*_*T*_ when a single searcher participates. See main text for discussion.(TIF)

S9 Fig The effect of rotational fluctuations is insensitive to the form of the binding-site interactions. To show that, we analyzed the same model, with the pairwise interactions decaying algebraically, rather than exponentially with distance: *U*(*θ*) = ∑[*r*(*θ*) − *r*_0_]^−3^. (A) Fraction of total attempted off-target pairings that reach *θ* = 0 with match probabilities, compared with that of the correct target. The initial angle limits contact and thus ensures that the majority of off-target pairings rapidly unbind, at the expense of some target pairings unbinding. (B) Mean unbinding time as a function of *N* for rotating rods that begin at *θ* = 0, and for constrained parallel rods that are allowed to unbind but not rotate, assuming a 1/*r*^3^ potential. Three accidental match probabilities are considered. Thermal fluctuations in angle destabilize pairings that have reached small angles and thus speeds up their unbinding.
(TIF)

S1 Text Supporting text. Derivation of the model. Monte Carlo simulations. Effect of parameter choices.
(PDF)
