# Supplementary figures and images for: Mechanisms of fast and stringent search in homologous pairing of double-stranded DNA
Source: PLoS Comput Biol. 2017 Mar 3;13(3):e1005421. doi: 10.1371/journal.pcbi.1005421 (PMC5360337; doi:10.1371/journal.pcbi.1005421)

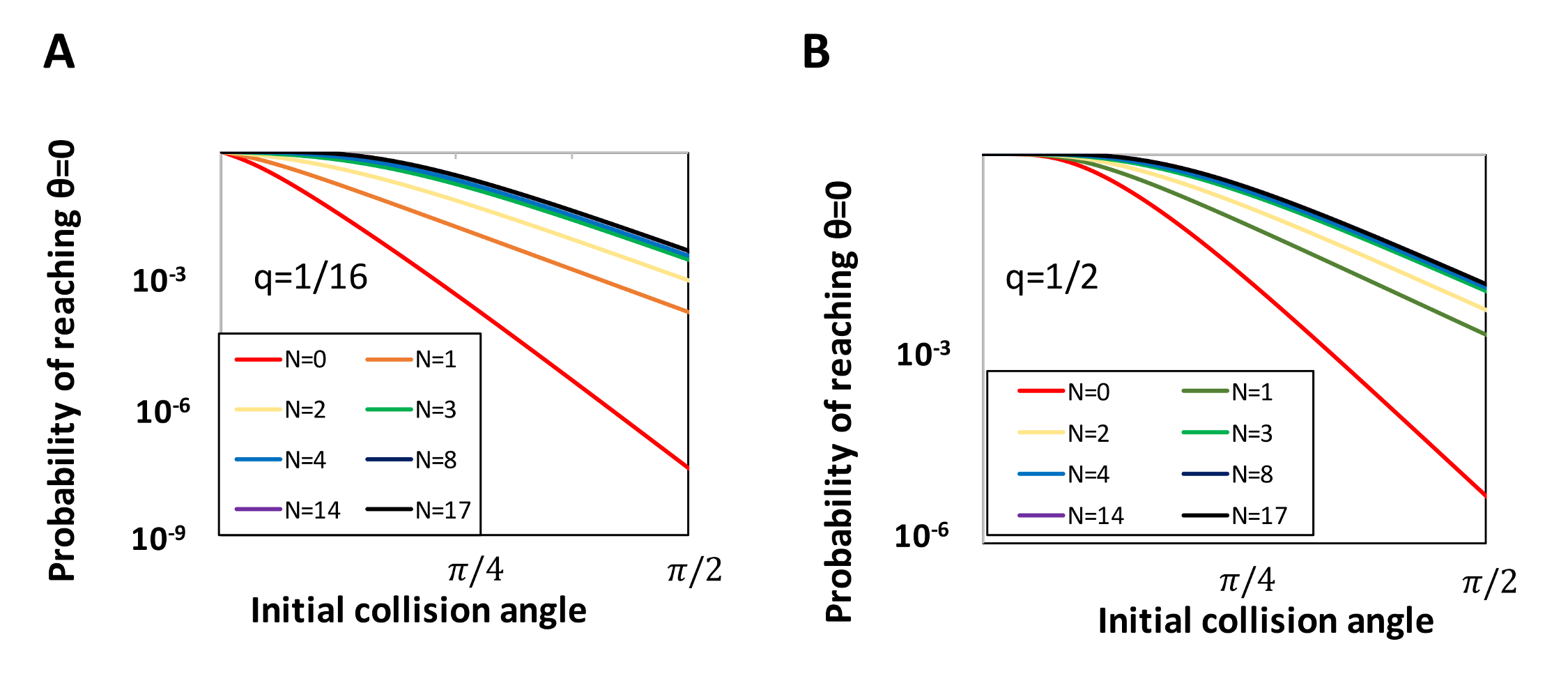

Supplement: S1 Fig — Same as Fig 1(B) of main text, but with (A) q = 1/16, and (B) q = 1/2. (TIF) [file pcbi.1005421.s001.tif]

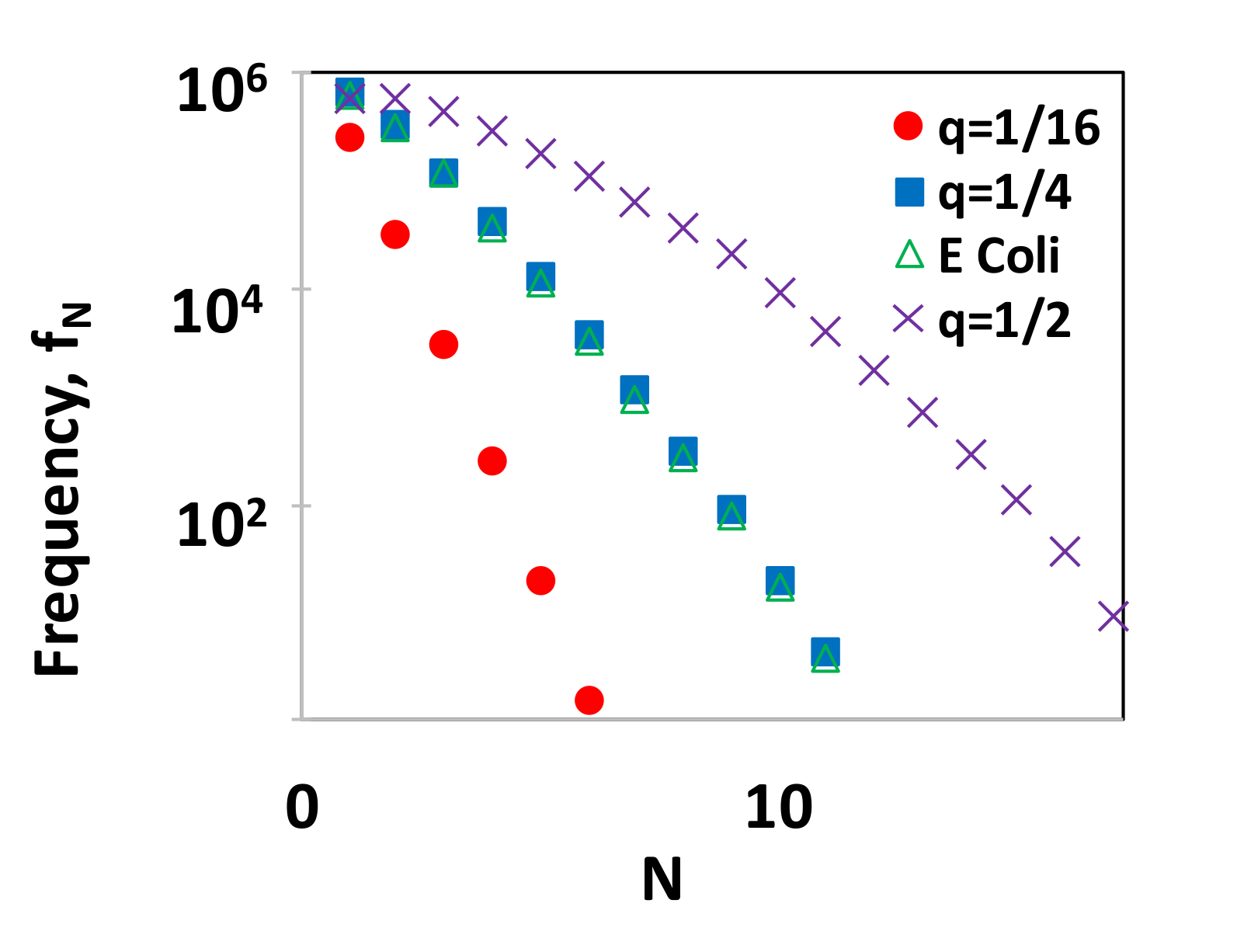

Supplement: S2 Fig — fN, the expected abundance of sequences that will match a given sequence at N continuous sites surrounding the collision site. Random genomes with different values of q are compared with the E. Coli genome. (TIF) [file pcbi.1005421.s002.tif]

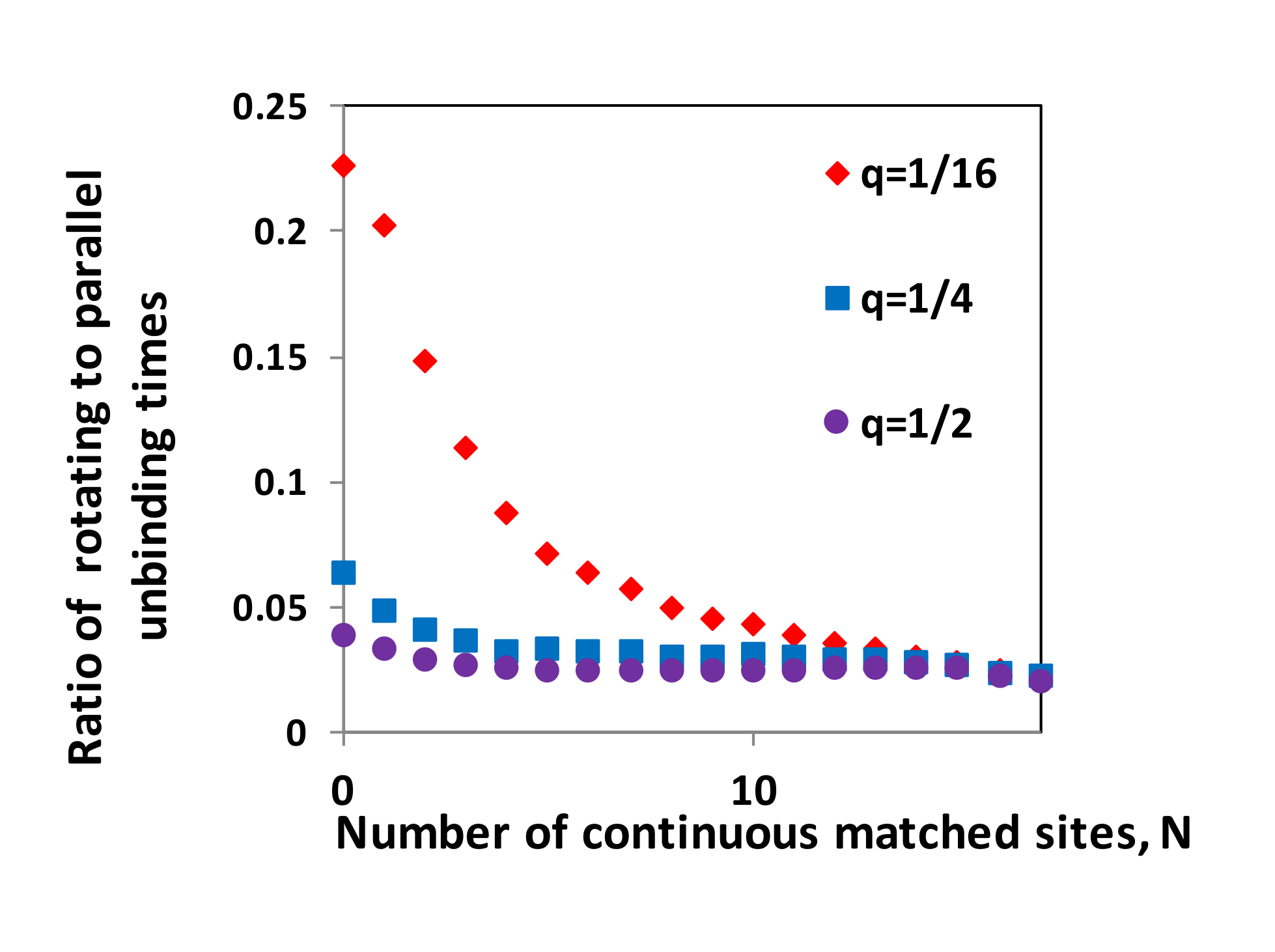

Supplement: S3 Fig — Ratio of mean unbinding time for rotating rods to mean unbinding time for parallel rods as a function of N, the number of continuous matched sites, assuming the rotating rods begin at the initial state of θ = 0, for several accidental match frequencies q. The effect of rotational fluctuations is enhanced when the number of matches between the two sequences increases and the free-energy profile becomes steeper. This can happen either by imposing larger number of matches (larger N) or by more frequent accidental matches (larger q). (TIF) [file pcbi.1005421.s003.tif]

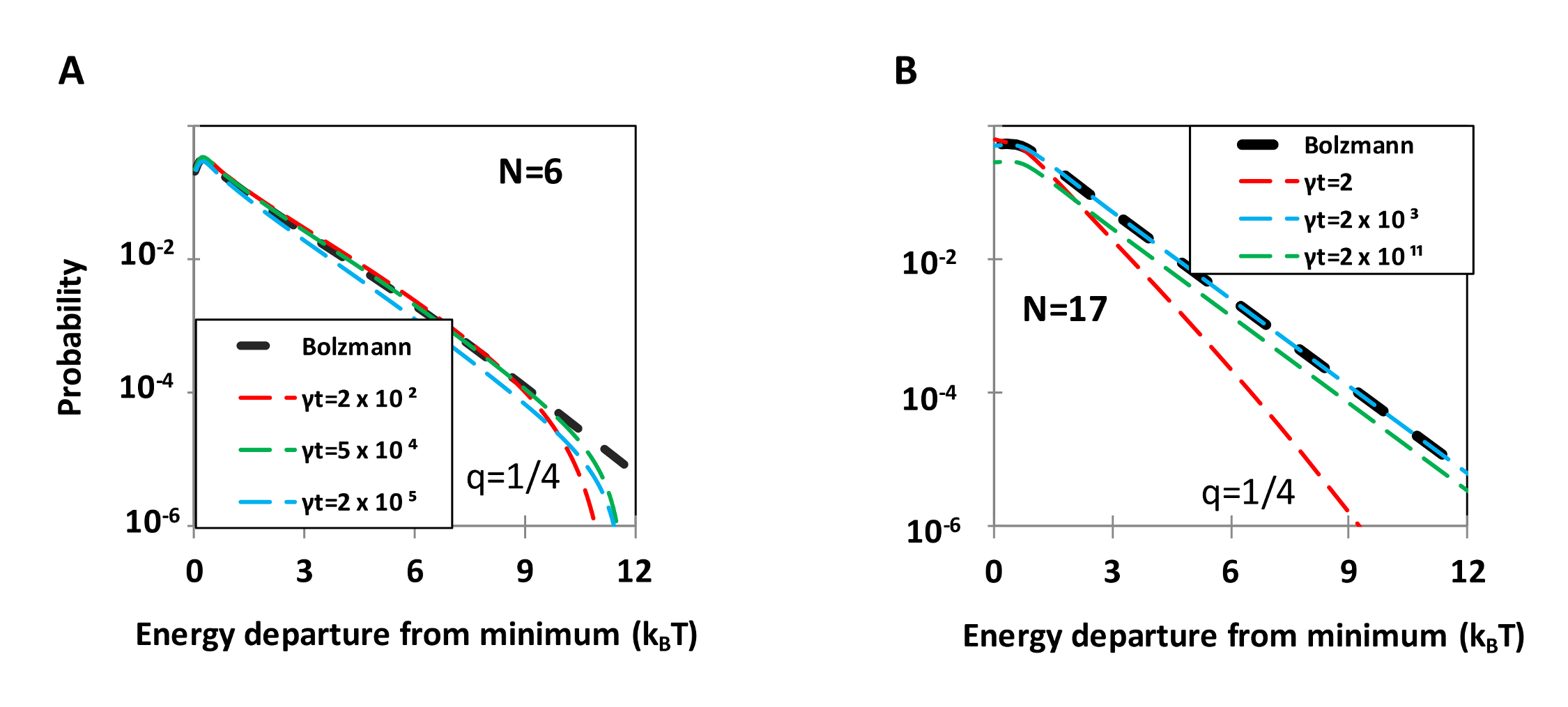

Supplement: S4 Fig — (A) Shows the probability of rotating to an angle with a certain departure in energy relative to the energy value at θ = 0 (x axis) at various times after a collision. At each time, the probability is averaged over 2000 randomly generated sequences with N = 6 continuous matches about the center and q = 1/4. This distribution is plotted alongside the Botlzmann distribution, which is averaged over the same 2000 sequences. (B) Same as (A) with N = 17. (TIF) [file pcbi.1005421.s004.tif]

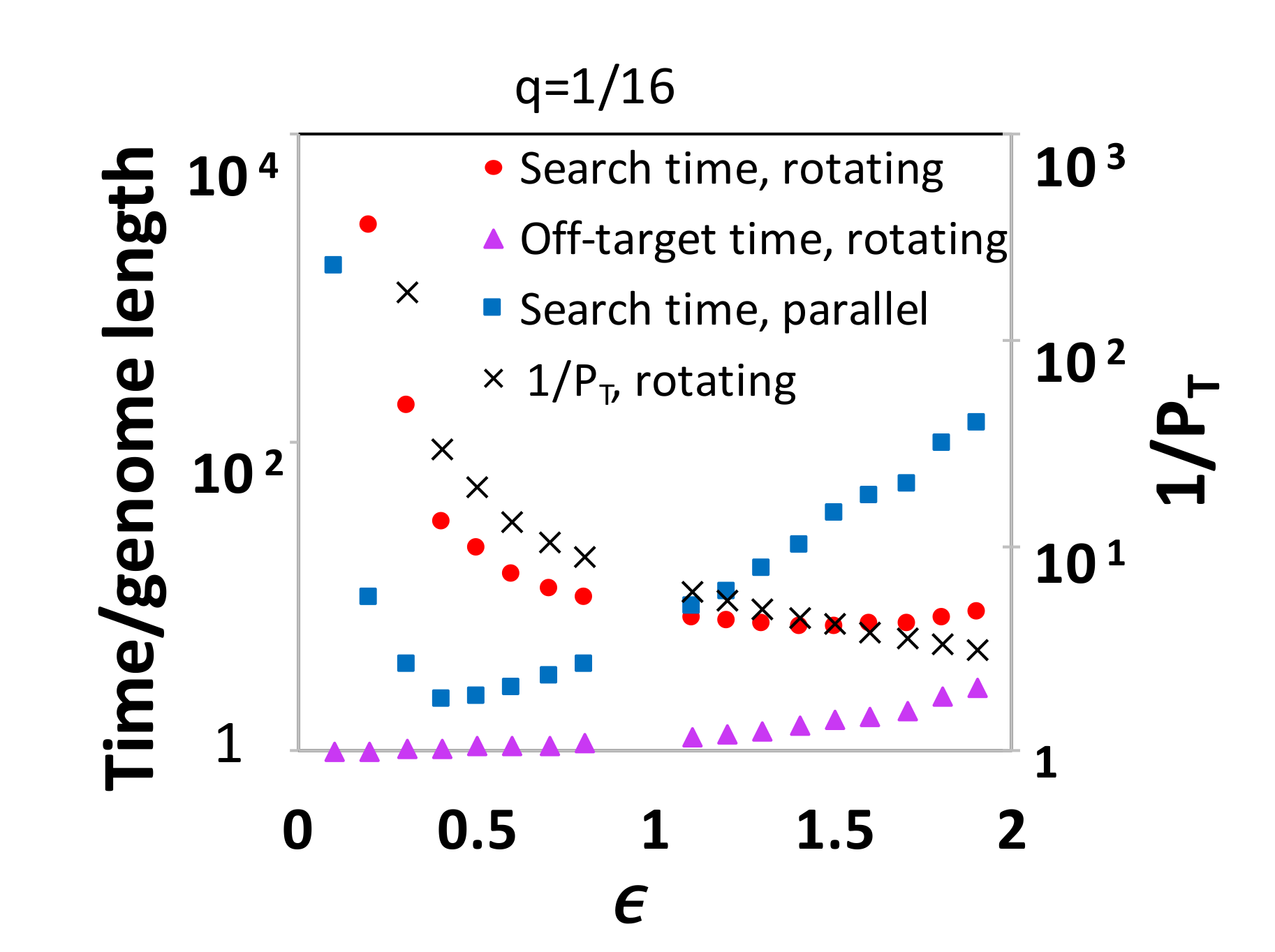

Supplement: S5 Fig — Off-target time and total search time for freely rotating rods, and search time for constrained parallel rods as a function of ϵ, the attractive energy per matched site in units of kB T. Collisions angles are assumed to be uniformly distributed in 3d between 0 and π/2 and q is set to 1/16. Values for 1/PT are shown on the right vertical axis. (TIF) [file pcbi.1005421.s005.tif]

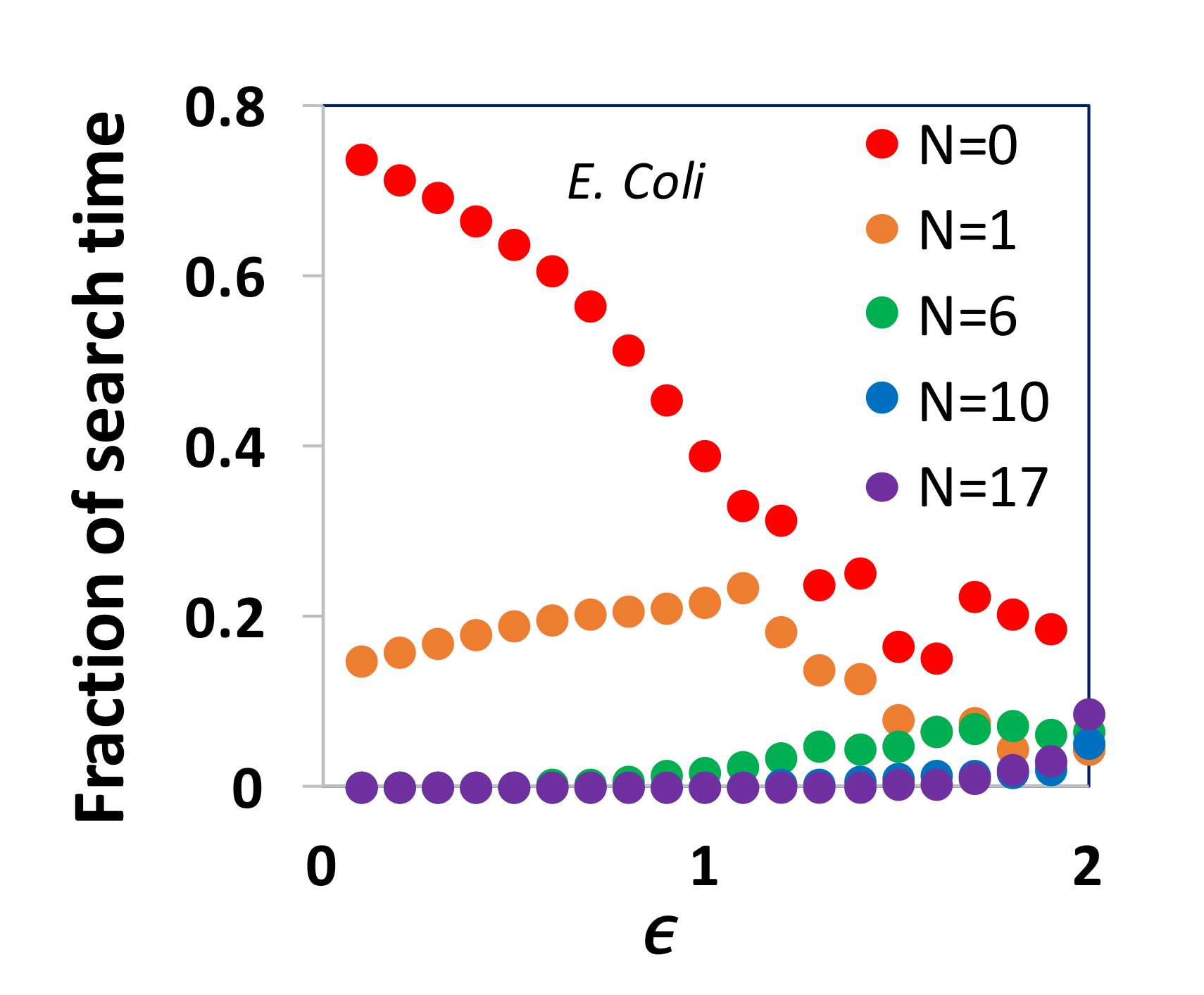

Supplement: S6 Fig — Fraction of search time spent interacting with off-target sequences with N continuous matches as a function of ϵ, the attractive energy per matched site. At low ϵ the search time is dominated by pairings with low N. But as ϵ increases, kinetic trapping becomes more problematic. Thus, the off-target interaction time becomes dominated by species with higher N when ϵ is large. Genome statistics from the E. Coli genome are assumed. (TIF) [file pcbi.1005421.s006.tif]

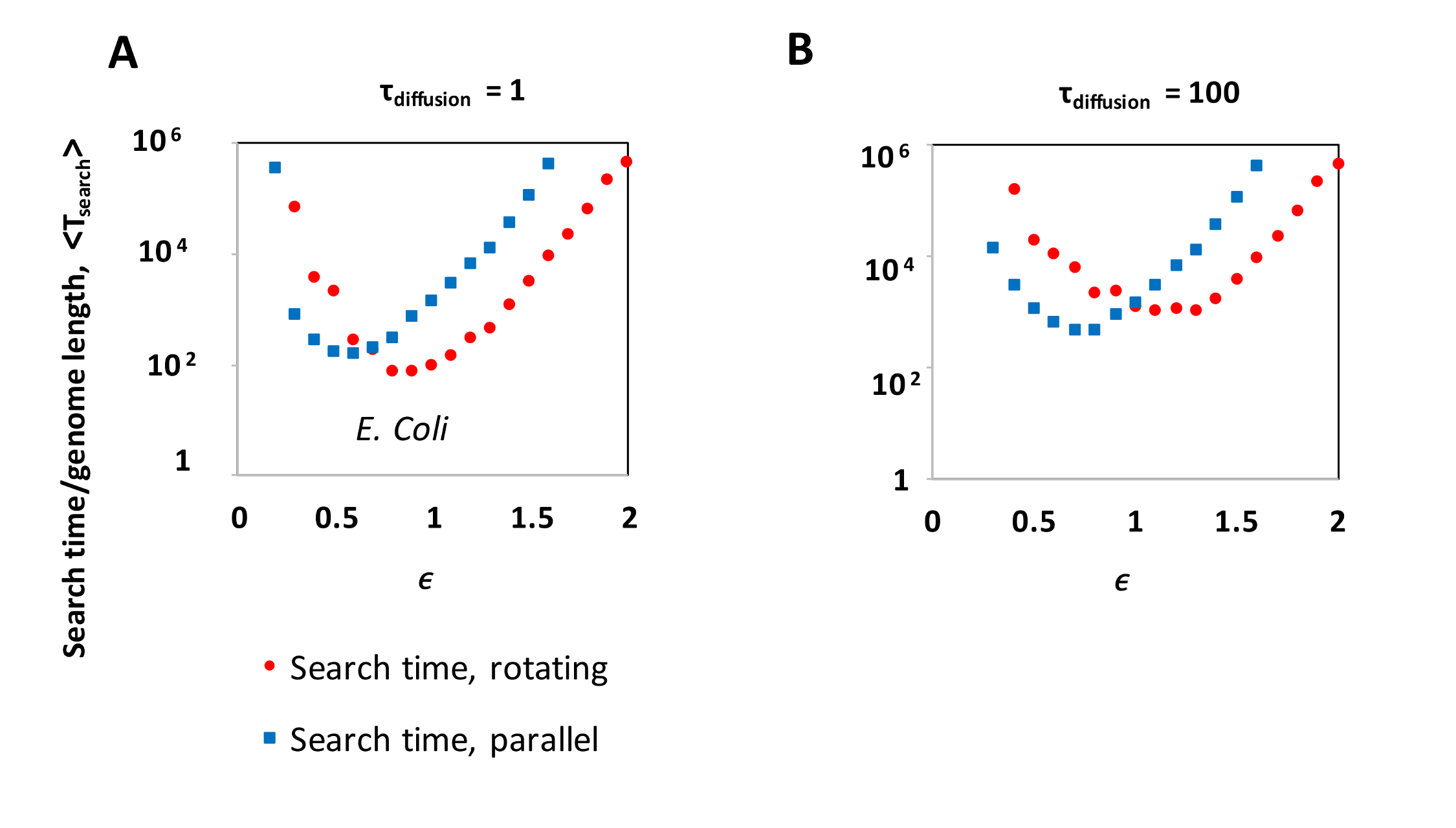

Supplement: S7 Fig — (A) Search time as a function of ϵ for freely rotating and constrained parallel rods assuming a diffusion time τoff of 1 (A) and 100 (B). Collision angles are averaged up to π/2 as in the main text, and genome statistics from the E. Coli genome are used. Increasing diffusion time penalizes the rotating system to a greater extent than the parallel system owing to the former’s low value of PT. Nonetheless, rotation is consistently beneficial above a minimum ϵ value, which increases along with τoff. (TIF) [file pcbi.1005421.s007.tif]

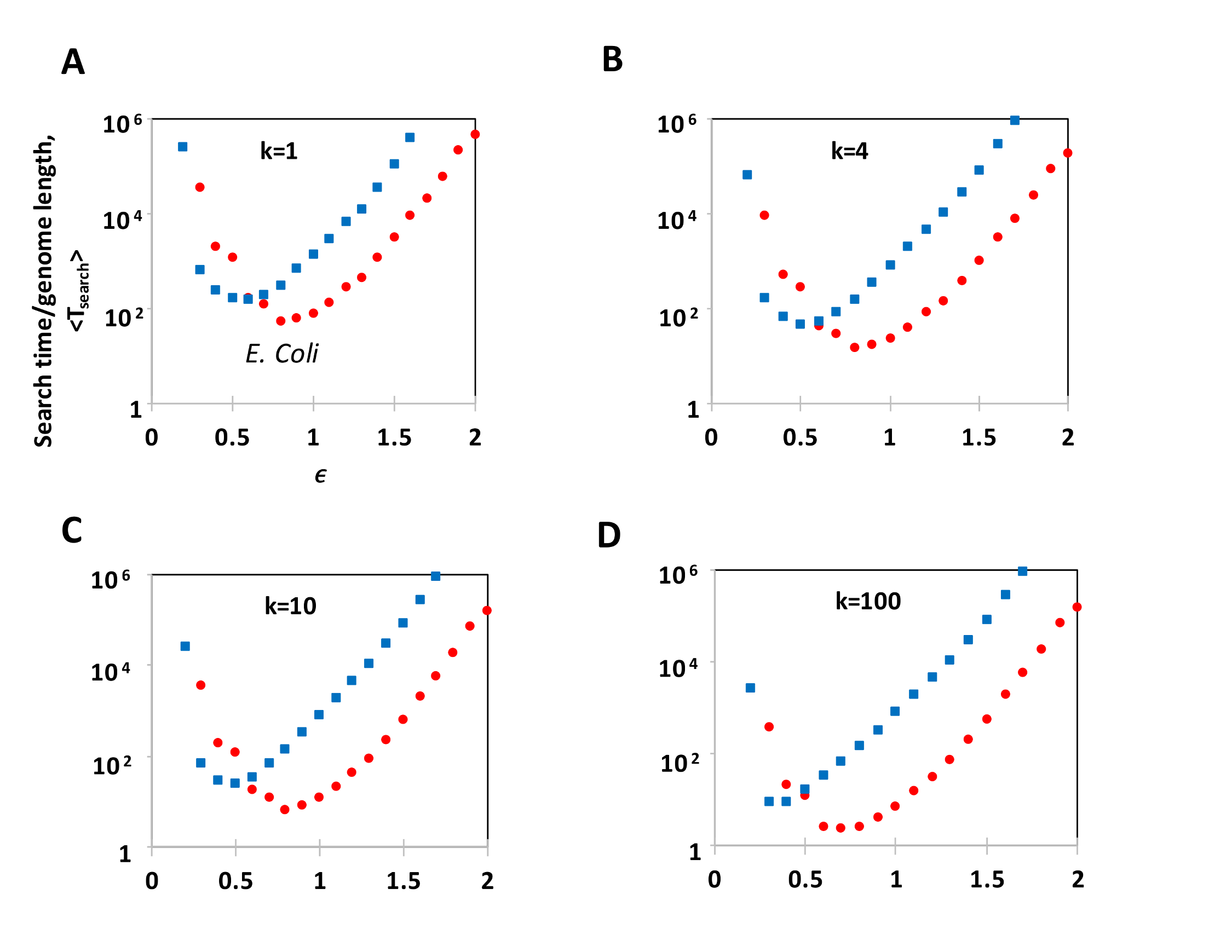

Supplement: S8 Fig — (A-D) The effect of k genomic segments searching in parallel on the search time, as a function of ϵ. At low ϵ, PT for a single searcher is very small due to the specificity requirement, and parallel searching is not useful. But at high energies, RH′ approaches 1 as k grows. Thus, both search times are decreased, particularly the rotating search time, which suffers from a worse PT when a single searcher participates. See main text for discussion. (TIF) [file pcbi.1005421.s008.tif]

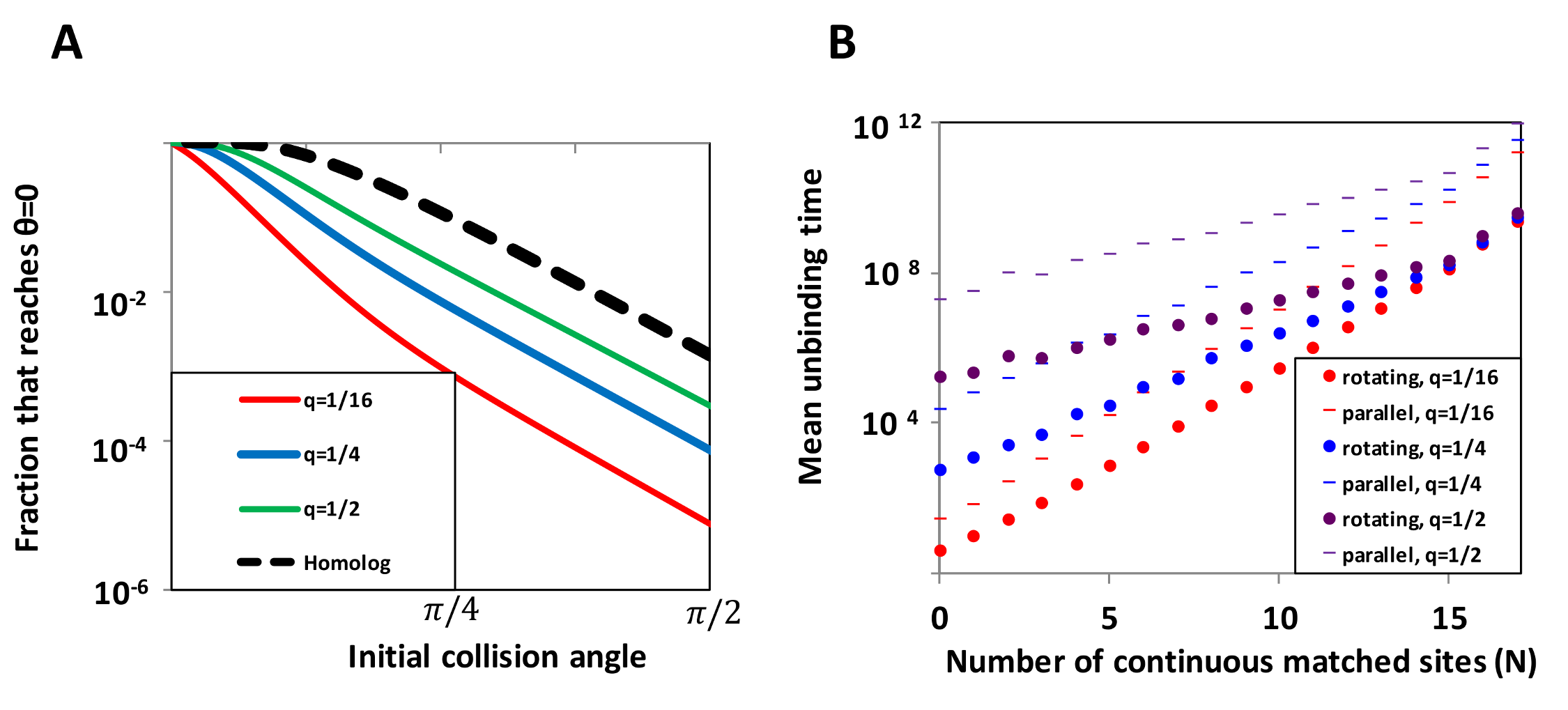

Supplement: S9 Fig — To show that, we analyzed the same model, with the pairwise interactions decaying algebraically, rather than exponentially with distance: U(θ) = ∑[r(θ) − r0]−3. (A) Fraction of total attempted off-target pairings that reach θ = 0 with match probabilities, compared with that of the correct target. The initial angle limits contact and thus ensures that the majority of off-target pairings rapidly unbind, at the expense of some target pairings unbinding. (B) Mean unbinding time as a function of N for rotating rods that begin at θ = 0, and for constrained parallel rods that are allowed to unbind but not rotate, assuming a 1/r3 potential. Three accidental match probabilities are considered. Thermal fluctuations in angle destabilize pairings that have reached small angles and thus speeds up their unbinding. (TIF) [file pcbi.1005421.s009.tif]
