# Supplementary material for: Mechanisms of fast and stringent search in homologous pairing of double-stranded DNA
Source: PLoS Comput Biol. 2017 Mar 3;13(3):e1005421. doi: 10.1371/journal.pcbi.1005421 (PMC5360337; doi:10.1371/journal.pcbi.1005421)
Supplement: S1 Text — Derivation of the model. Monte Carlo simulations. Effect of parameter choices. (PDF) [file pcbi.1005421.s010.pdf]

Supporting Information

Bitran et al.

I. METHODS

A. Rigid rod approximation

In our model, we consider the interaction between a pair of rigid rods, each of which corresponds to a DNA fragment over the short region that immediately flanks a collision site. The binding sites on the rods are separated by 3.4 nm, on the assumption that two dsDNA molecules interact roughly once per helical turn. The total length of the rods is 17 binding sites, or 57.8 nm, which is comparable to measured values for the persistence length of dsDNA [**?**]. We assume, for simplicity, that the rods always collide at their centers of mass (which corresponds to the 8th binding site), and that, following a collision, their centers of mass are separated by 1 nm. The collision angle, θ, is assumed to vary from 0 to $\pi/2$ (all collision angles above $\pi/2$ are treated as a collision between a different pair of sequences with collision angle $\pi - \theta$).

B. Sequence assignment

Each rigid rod is assigned a sequence. The sequence labels are numbers ranging from 1 to $1/q$, where q is the frequency of accidental matches in the system. An important variable in this system is N, the number of continuous matched sites surrounding the collision site between the rods. When the number N of matched sites flanking the collision site is given, we assign the same labels to sites in registration, symmetrically distributed about the collision site. We also impose the restriction that the two sites immediately flanking the N matched sites are guaranteed *not* to match. Finally, sites beyond those $N + 2$ may match with a probability given by q. The exception is $N = 0$, where the only constraint on the sequences is that the collision site does not match.

C. Discrete-state approximation

To simplify the analysis, we discretize the bound states by dividing the range of angles $\theta \in [0, \pi/2]$ into 201 equally spaced angles, enumerated by $k = 0 .. 200$, and assume that rotations occur only between adjacent states. We define $P_{k,i}(t)$ to be the probability that a collision at an initial angle corresponding to state i leads to state k at time t. $P_{k,i}(t)$ obeys the master equation:

$$\frac{d}{dt}P_{k,i}(t) = M_{k,k-1}P_{k-1,i}(t) + M_{k,k+1}P_{k+1,i}(t) - (M_{k+1,k} + M_{k-1,k} + M_{A,k})P_{k,i}(t) \ , \quad (1)$$

where the transition matrix elements of the form $M_{k,k'}$ give the probability per unit time of transitioning from state k' to k. These elements are given by $M_{k,k'} = \gamma \min(1, \exp(-(U(k') - U(k))/k_BT))\delta_{|k-k'|,1}$ where γ sets the time scale for rotation, $U(k)$ is the total energy of state k, k_B is the Boltzmann constant, and T is the temperature. Meanwhile, $M_{A,k}$ denotes the probability per unit time of unbinding. Choosing units in which the unbinding rate for pairs with no interaction is 1, this is given by $M_{A,k} = \min(1, \exp(U(k)/k_BT)$. The formal solution to the master equation is

$$P_{k,i}(t) = \sum_j C_{ji}V_{kj}e^{\lambda_j t} \ , \quad (2)$$

where λ_j are the eigenvalues of the matrix M, $\mathbf{V}_j$ are the corresponding eigenvectors, and the coefficient matrix $\mathbf{C}$ is determined by the initial state. Their numerical values, given a choice of pairwise interaction energies and their parameters, was determined using the built-in function `eig` in MATLAB (Mathworks, Natwick MA). Unless otherwise noted, calculations were done on 2000-3000 random sequences with N continuous matched sites, and a probability q of matching otherwise.

D. Energy

We assume that the energy between any pair of matched sites is attractive over short distances and decays exponentially with distance. This may model an electrostatic interaction subject to Debye screening. The energy due to two matched sites is thus $-\epsilon e^{-(r(\theta)-r_0)/\lambda_D}$, where $r(\theta)$ is the distance between the matched sites, which depends on the rods' angle, r_0 is the distance between two sites that are in register when the rods are bound and parallel, λ_D is the Debye screening length, and ϵ is the absolute value of the attractive energy per matched site, in units of k_BT. Meanwhile, we assume mismatched sites experience no interaction. Thus, the total energy between the pair of rods is given by

$$U(\theta) = \sum_{\text{matched sites}} -\epsilon e^{-(r(\theta)-r_0)/\lambda_D} \quad (3)$$

where the sums run over every pair of matched sites, regardless of whether or not the sites are in registration (for example, if site 1 on one rod matches site 1 and site 5 on the other, then the sum includes both of these interactions). However, the sums are dominated by the pairs that are in registration, as these are closest to each other. We assume that the separation between the rods' centers of mass is $r_0 = 1$ nm. The decay length λ_D is also set to 1 nm. Modifying the value of these length scales

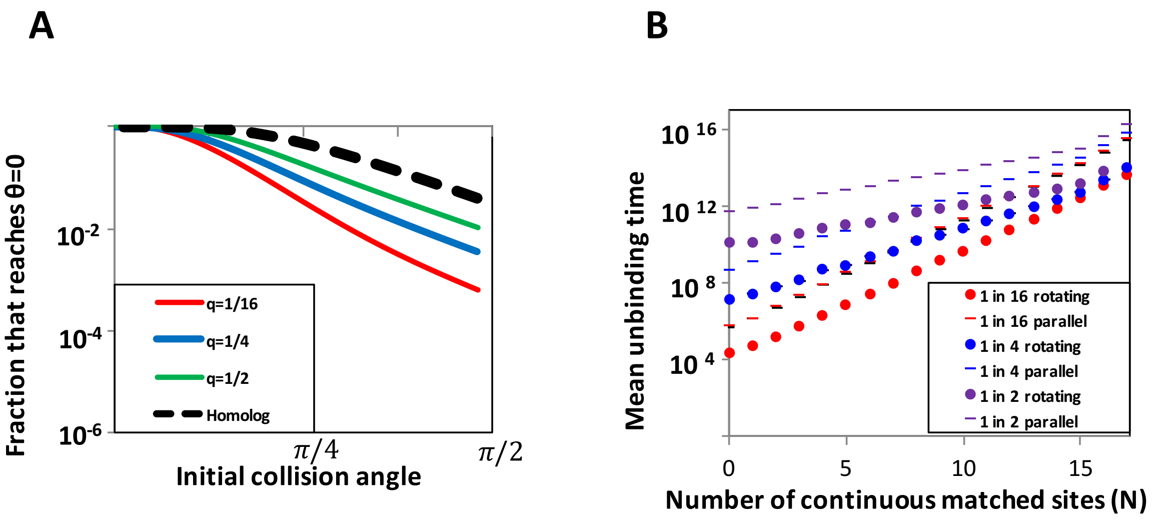

Supporting Figure S10: **The effect of nonspecific interactions between mismatched sites.** (A) Fraction of total off-target and on-target pairings that reach $\theta = 0$ as a function of collision angle, and (B) Mean unbinding time as a function of N for rotating rods that begin at $\theta = 0$ and for constrained parallel rods that are allowed to unbind but not to rotate, assuming an exponentially decaying attractive potential with energy between aligned matched sites of $2k_BT$, and energy between aligned mismatched sites of $0.5k_BT$. Three accidental match probabilities are considered.

does not affect the system behavior so long as the ratio between the decay length and the binding site spacing and the ratio between the decay length and the rods' separation remain constant.

In a particular system mismatched sites may also experience an attractive interaction. So long as this nonspecific interaction is weak, the essential results of this paper are expected to remain mostly unchanged. However, such attractive interactions render collisions at finite angles less effective at filtering out mismatches, and increase the time for unbinding (Supporting Figure S10). On the other hand, strong attraction between mismatched sites is detrimental, as it extends the time required for kinetic proofreading beyond physiological timescales.

E. Mean time to unbinding

Of key importance to our results below are the mean times from collision to unbinding, and its dependence on the number N of consecutive matching sites around the collision point. We define the vector $\boldsymbol{\tau}_N$ whose elements $\tau_N(i)$ are mean times to unbinding starting from state i, and compute it by solving numerically the equation [?]

$$-1 = M^T \tau_N, \tag{4}$$

where M^T is the transpose of the transition matrix M.

F. Probability of arriving at a parallel configuration

In the text, we are often interested in the probability that a pairing will reach $\theta = 0$. To solve for this probability, we determine the eigenvectors and eigenvalues of M, the transition matrix in equation (1), but with the state corresponding to $\theta = 0$, set as an absorbing boundary (in addition to the absorbing boundary A which accounts for unbound molecules). Two of the eigenvectors of the matrix M therefore correspond to the two absorbing states, both with eigenvalues that equal zero. For a given initial state i, equation (2) gives the time evolution of the system. The probability of reaching each absorbing state j from the initial state i is then given by the coefficient C_{ji} of the corresponding eigenvector.

G. Frequency of pairings with N continuous matches

In this work, we often compute the expected number of sequences in the genome that match a given sequence in N continuous sites about the center. As always, we assume the rods are 17 sites long. To evaluate this frequency for $N > 0$, we assume that the distinct types of sites are randomly distributed throughout the genome, and that the collision site corresponds to the center of the matched region. The multiplicity of registrations is accounted for by a combinatorial prefactor R_N, which has values $R_1 = 1$, $R_2 = 2$, ...$R_9 = 9$, $R_{10} = 8$,...$R_{17} = 1$. The frequency of sequences that match at N continuous

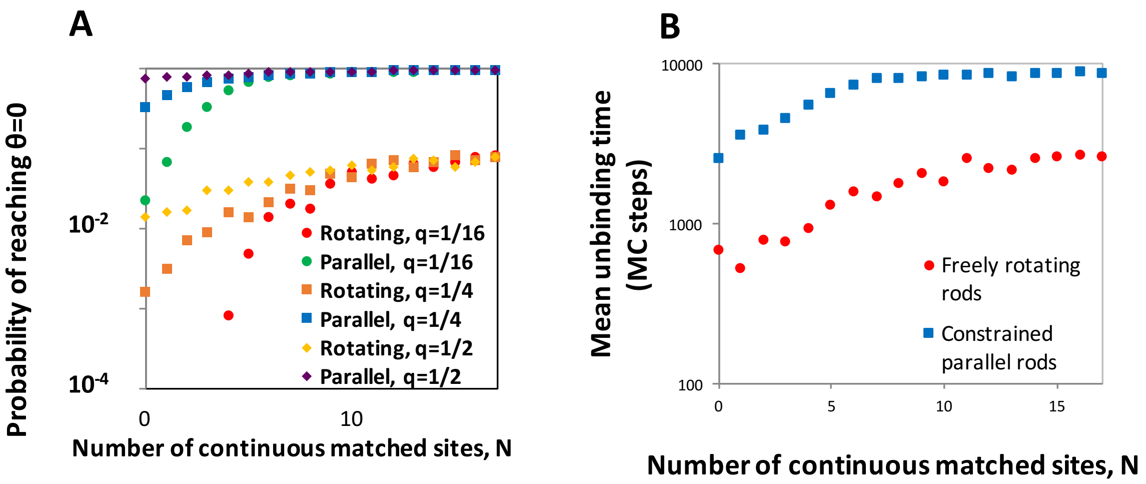

Supporting Figure S11: **Monte Carlo simulations.** (A) Monte Carlo simulation results for the probability of reaching a small angle as a function of N, the number of continuous matches surrounding the center site. Three probabilities q for accidental homology beyond N are considered. We also show the probability of remaining bound at a small distance for 10 MC steps if the rods are constrained to lie in parallel. (B). Monte Carlo simulation results for mean unbinding times in MC steps, with $q = 1/4$. Rods are allowed to freely rotate following a collision at $\theta = 0$, or constrained to lie in parallel.

sites about the center given accidental match frequency q is then given by

$$f_N = R_N q^N (1 - q)^2 \tag{5}$$

for $N > 0$, and

$$f_0 = 1 - q\ . \tag{6}$$

H. *E. Coli* genome statistics

In various sections of this paper, we consider the average number of sequences in the *E. Coli* genome that match a test sequence in N continuous matches about the center. To compute this quantity, we randomly select 10,000 noncontinuous sequences in the *E. Coli* genome, where the bases constituting the sequence are separated by 10 bp (roughly one helical turn of DNA, assuming interaction sites occur once per turn). Each such sequence is 17 bp long. We test each such sequence against every other 17 bp sequence in the genome, each of which is also comprised of base pairs separated by 10 bp, and compute N about the center. We then average the resulting frequencies f_N among the 10,000 random test sequences. The results, plotted in supplementary figure 2B, closely resemble those for a random genome with $q = 1/4$.

Parallel rods

Throughout the work, we compare our system of rotating rods to a reference system, in which the rods are constrained to lie in parallel. The parallel rods are also 17 sites long, and they have the same sequence assignment and energy form as the rotating rods. Because of the rotational constraint, the distance between every pair of corresponding sites is fixed at $r_0 = 1$ nm. In this system, the rods can only unbind with probability per unit time $M_A = \exp(U/kT)$, and so the probability that the rods are bound at time t, $P_{\mathrm{parallel}}(t)$, satisfies

$$\frac{d}{dt} P_{\mathrm{parallel}}(t) = -M_A P_{\mathrm{parallel}}(t) \tag{7}$$

which, given the initial condition $P_{parallel}(0) = 1$, has solution

$$P_{\mathrm{parallel}}(t) = e^{-M_A t}\ . \tag{8}$$

The mean unbinding time for the parallel rods is simply the reciprocal of the unbinding probability per unit time, or

$$\langle \tau_{\mathrm{parallel}} \rangle = \frac{1}{M_A}\ . \tag{9}$$

II. MONTE CARLO SIMULATIONS

As an alternative to the discrete-state model described in the main text, we performed Monte Carlo simulations in which we model the interaction between two rigid rods with N continuous matched sites. In each simulation, rods collide at a random angle chosen uniformly over the unit sphere, and are allowed to freely rotate or diffuse in 3 dimensions using standard Monte Carlo transition probabilities. They may also collide up to 1 nm off registration. Finally, the initial distance between the rods' centers of mass varies randomly with a minimum value of 1 nm. Using these simulations, we sought to test the

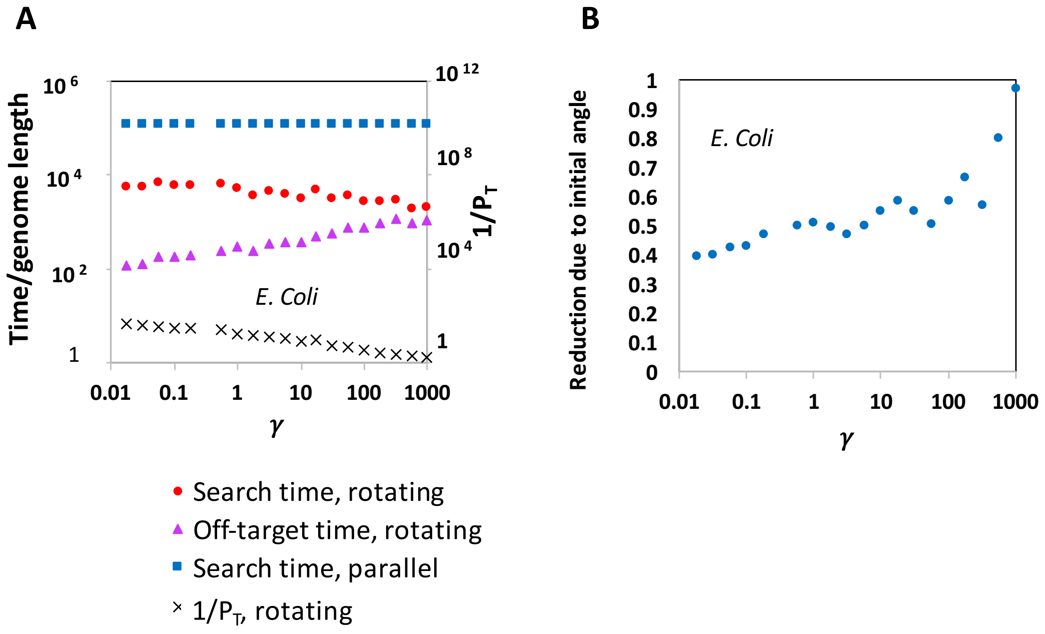

Supporting Figure S12: **Effect of γ on search time** (A) Shows the search time for rotating and parallel rods, off-target time for rotating rods, and $1/P_T$ for rotating rods as a function of γ, the ratio of the timescale for rotational fluctuations to the timescale for unbinding. Collisions are assumed to be uniformly distributed up to $\pi/2$ as in the main text, and genome statistics from the *E. Coli* genome are used. (B) Ratio of the homology search time if angles are uniformly distributed from 0 to $\pi/2$, to the search time if collisions are constrained to occur in parallel.

two key predictions of our master equation approach: (1) Rotation into small angles is homology-dependent, and (2) Thermal fluctuations in angle speed up unbinding.

To test the first prediction, we ran simulations in which the rods interact until either of the following two outcomes occur, at which point the simulation ends: (i) they reach a sufficiently small angle and distance and dwell there for 10 MC steps, or (ii) they separate by 10 nm, at which point they are assumed to have irreversibly unbound. We then measure the frequency at which the first outcome occurs. The results are shown in Fig. S11A for various accidental match probabilities q. As with our main model, these results show that rotation into parallel is homology-dependent. Numerical differences between the two approaches can be attributed to different dimensionality of the rotational degree of freedom, as well as to fluctuations in the distance between the molecules.

Next, we corroborated the result that rotational fluctuations reduce unbinding time, by comparing simulations of the full model with simulation of a model where the two rods are always parallel. The results are shown in Fig. S11B for $q = 1/4$. Indeed, the unbinding times for the freely rotating rods are consistently smaller by about an order of magnitude, further suggesting that thermal fluctuations in angle may play an important role in destabilizing pairings. We truncate all simulations at 10,000 MC steps, so that they do not take too long to run. This explains the plateau at large N values. But we would expect this pattern to continue if we did not truncate the simulations.

III. EFFECTS OF PARAMETER CHOICES ON RESULTS

In Fig. 4A of the main text, we considered the search time as a function of energy, assuming with *E. Coli* genome statistics (which resembles a random genome with $q = 1/4$). Fig. S5 shows the same search time for $q = 1/16$. While this result qualitatively resembles that for the bacterial genome, we note that rotation no longer yields the global search time minimum. When $q = 1/16$, this minimum belongs to the parallel system at low ϵ. This is because accidental matches are now much rarer. As a result, kinetic trapping is less of a problem, so the rotating system no longer benefits as much from reduced kinetic trapping relative to the parallel system. Furthermore, this lower accidental match probability implies a larger energy gap between matches and the nearest mismatch. This means that the parallel system can afford to work at lower ϵ values without suffering from a poor P_T. This result highlights the fact that rotation is most beneficial when there is significant kinetic trapping, and a significant speed-stringency tradeoff.

In Fig. S12, we consider how varying γ, the ratio between the characteristic time for rotation and the characteristic time for unbinding, affects the homology search time. We plot $\tau_{\rm off}$, the homology search time, and $1/P_T$ for a wide range of γ values. We also include the homology search time for constrained parallel rods for reference, which is of course independent of γ. Increase in γ represent a tradeoff between a decreasing value $1/P_T$ and an increasing value of $\tau_{\rm off}$. When $\gamma \ll 1$, rotation is slow compared to unbinding, and few pairings reach small angles. This results in a low $\tau_{\rm off}$ due to minimal kinetic trapping, but also a low P_T. As γ increases, rotation becomes faster so more pairings reach small angles. This has the reverse effect on $\tau_{\rm off}$ and P_T. As a result of this tradeoff, the overall homology search time is relatively insensitive to γ, decreasing only slightly as γ grows large. This slight decrease is due to the fact that $\tau_{\rm off}$ grows more slowly with γ than $1/P_T$ decreases.

Thermal fluctuations in angle following rotation into parallel play a role in suppressing the increase in $\tau_{\rm off}$, as fluctuations become faster and thus more destabilizing when γ is large. But the relative constancy of the homology search time as a function of γ ensures that rotation decreases the search time relative to the parallel system over a wide range of γ values. Moreover, given that $1/P_T$ does not exceed 80 over the range of γ values considered, it may be feasible to vastly parallelize the search and thus further reduce the search time in the rotating system relative to the constrained parallel reference system. But this may not be as useful for γ values smaller than those considered here, at which point $1/P_T$ would become very large and many searchers would be required for a significant improvement. Over the range of γ values considered, rotation is always beneficial. But we anticipate that reducing γ so that it is many orders of magnitude smaller than the minimum value considered here would freeze out the rotational degree of freedom altogether, and render rotation useless.

The value of γ also affects the degree to which the initial collision angle θ_0 benefits the search time. In Fig. S12B we plot the ratio of the homology search time if angles are uniformly distributed from 0 to $\pi/2$, to the search time if collisions are constrained to occur in parallel (but subsequent rotational fluctuations are permitted). The lower this ratio, the more the system benefits from colliding at nonzero angles. We see that the initial angle is most beneficial at low values of γ. This is because when γ is small, rotational fluctuations are slow enough to ensure that most mismatched pairings do not reach the deeply bound state. Of course, decreasing γ also decreases P_T, but not to the same extent as it decreases $\tau_{\rm off}$. Conversely, high values of γ lead to a significant number of mismatched pairings rotating into the parallel, deeply bound state even if the collision angle is nonzero. Thus, the collision angle is less beneficial. Overall, the search time reduction due to the collision angle is never greater than ~ 0.4. Thus, the initial angle contributes a significantly smaller benefit than subsequent rotational fluctuations.
